# Differentially expressed gene profiles and associated ceRNA network in ATG7-Deficient lens epithelial cells under oxidative stress

**DOI:** 10.3389/fgene.2022.1088943

**Published:** 2022-12-07

**Authors:** Hongyu Li, Lixiong Gao, Jinlin Du, Tianju Ma, Zi Ye, Zhaohui Li

**Affiliations:** ^1^ Medical School of Chinese PLA, Beijing, China; ^2^ Senior Department of Ophthalmology, The Third Medical Center of PLA General Hospital, Beijing, China

**Keywords:** cataract, oxidative stress, autophagy, lens epithelial cells, differentially expressed genes (DEG), competing endogenous RNAs (ceRNA)

## Abstract

Oxidation is an essential factor during cataract development. Autophagy, usually a cytoprotective process, is always found elevated in lens epithelial cells under oxidation, yet its roles and associated molecular mechanisms under such circumstances are rarely elucidated. Herein, we extracted and re-analyzed the RNA sequencing data of the GSE161701 dataset from the Gene Expression Omnibus database to identify the differentially expressed mRNAs and lncRNAs by using the R package “DESeq2”. Further analyses of gene ontology and KEGG enrichment were implemented *via* the packages “clusterProfiler” and “enrichplot”. We found that after the knockout of *ATG7*, differentially expressed genes were more associated with hemopoiesis, vasculature development, axonogenesis, and hypoxia regulation. When stimulated with H_2_O_2_, LECs displayed a gene expression profile correlating with apoptotic and proliferative pathways, such as the MAPK signaling pathway and FoxO signaling pathway. The differentially expressed gene profiles of the two types of LECs (wild type and ATG7 deficient) under oxidation were distinct to a large extent. Furthermore, 1,341 up-regulated and 1912 down-regulated differential mRNAs and 263 up-regulated and 336 down-regulated differential lncRNAs between these two types of LECs subjected to H_2_O_2_ were detected, among which 292 mRNAs and 24 lncRNAs possibly interacted with ten cataract-related miRNAs. A competing endogenous lncRNA-miRNA-mRNA network based on such interactions was finally constructed.

## Background

Cataract, a common cause of vision loss, results from the gradual opacification of the lens, mostly at an elderly age. It has been recognized that risk factors such as aging, diabetes mellitus, ultraviolet B exposure, long-term use of corticosteroids, and smoking can contribute to the development of cataract. (Truscott, 2005). Physiologically, the lens epithelial cells (LECs) residing under the anterior capsule of the lens continuously proliferate and differentiate into the elongated fiber cells to form the compacted nuclear mass of the lens throughout its lifespan. These normal activities of LECs guarantee the homeostasis and transparency of the lens. And any disturbance to the LECs activities can result in cataractogenesis. A marked reduction of the lens epithelial cell density is observed in advanced senile cataract. (Tseng et al., 1994). With the aging of the lens, the LECs demonstrate a remarked increase of apoptosis in a time-dependent manner, and the experimentally induced apoptosis of LECs can give rise to the development of cataracts. (Li et al., 1995; Zhang et al., 2010). Moreover, there is a relatively high level of apoptosis of LECs in diabetes-induced cataracts. (Takamura et al., 2003; Xie et al., 2022). However, the molecular mechanisms of cataract development are not fully elucidated.

Oxidative stress is a prominent and critical factor for cataractogenesis. Generally, the metabolically produced reactive oxygen species (ROS), such as hydrogen peroxide (H_2_O_2_) and hydroxyl radical, can be reduced by the antioxidants like reduced glutathione (GGH), thus maintaining a stable and balanced redox environment in LECs. (Giblin, 2000). Any disturbance to this balance, whether due to aging, ultraviolet exposure, or some other factors, will result in redundancy of free radicals, which in turn contribute to cataract development. (Yildirim et al., 2009; Pescosolido et al., 2016; Hsueh and Chen, 2022). The lens proteins, such as crystallins, are found oxidized and aggregated in the nucleus of the lens and therefore scatter the light during the development of cataract. (Cobb and Petrash, 2002; Vetter et al., 2020; Hanafy and Cave, 2021). Of note, the most metabolically-active LECs are more susceptible to oxidative radicals. Excessive ROS can incur the apoptosis of LECs by targeting the DNA, membrane proteins, and many other constitutive components (Long et al., 2004; Cui et al., 2012), while antioxidants administration can ameliorate the H_2_O_2_-induced apoptosis of LECs(Zhou et al., 2016; Bai et al., 2017). Nevertheless, the exact and comprehensive molecular mechanisms of LECs apoptosis and the ensuing cataract under oxidative stress are, to our knowledge, not completely elaborated.

Autophagy (here referred specifically to macroautophagy) is an evolutionally conserved, catabolic process across a variety of species, which can break down the dysfunctional or unneeded macromolecules and membrane-coated organelles in the cytoplasm to recycle the necessary building substrates. It entails the formation of autophagosomes, their fusion with lysosomes, and the degradation of the inside components, each phase involving certain critical proteins, such as BECN1, LC3B, and ATG7. Although it is controversial whether autophagy is involved in the formation of the organelle-free zone during lens development (Matsui et al., 2006; Nishida et al., 2009; Morishita et al., 2013; Tu et al., 2021; Gheyas et al., 2022), the development of cataract is closely associated with dysfunctional autophagy. (Morishita, Eguchi, Kimura, Sasaki, Sakamaki, Robinson, Sasaki and Mizushima, 2013; Ping et al., 2021). A recent study revealed that rapamycin-induced autophagy can alleviate the level of ROS in LECs cultured in high glucose. (Liu J. et al., 2020). Furthermore, an elevated autography accompanied reduced apoptosis in H_2_O_2_-treated LECs(Han et al., 2021), and the overexpression of *ATG4a* in LECs can mitigate the apoptosis of cells. (Yan et al., 2020). It may suggest a cytoprotective role of autophagy toward LECs under oxidative stress by regulating cell apoptosis, which is quite contradictory to the findings of Huang J et al., whose study revealed autophagy-facilitated apoptosis. (Huang et al., 2022). Therefore, the detailed molecular mechanisms concerning the roles of autophagy in oxidative stress need further explored.

Non-coding RNAs (ncRNAs) refer to a considerable amount of transcriptomes without protein-coding function, which can be simply grouped into long non-coding RNAs (lncRNAs), small RNAs like microRNAs (miRNAs), transfer RNAs (tRNAs) and ribosomal RNAs (rRNAs) based on their length and function. Over the past few years, ncRNAs, especially lncRNAs and miRNAs, are found to play a vital role in the development of a variety of diseases, including cataracts. Recent studies showed that the elevated miR-23b-3p and miR-34a expressions could promote the apoptosis of LECs under the oxidative stress state while overexpression of miR-124 reduced the apoptosis of H_2_O_2_-treated LECs. (Fan et al., 2017; Gu, 2018; Zhou et al., 2019; Zhang et al., 2020). Moreover, lncRNA-H19 was up-regulated in cataract tissue and its knockdown could accelerate apoptosis of LECs under oxidative stress, and lncRNA TUG1 can promote the apoptosis of H_2_O_2_-treated LECs *via* targeting the miR-196a-5p. (Liu et al., 2018; Shen and Zhou, 2021). Although there is growing evidence of ncRNAs involved in pathogenic processes during cataract development, the comprehensive profile of ncRNAs and the interaction network concerning cataractogenesis have not been reported.

Autophagy requires the elongation of the phagophore at the beginning, during which ATG7 is critical for the formation and activation of two important complexes, ATG12-ATG5 complex and LC3-PE complex (Rubinsztein et al., 2012). Ablation of ATG7 is thought to completely block the elongation of the phagophore, thus abolishing the autophagy activity. Herein, we selected the LECs knocked out of *ATG7* to abolish the autophagy process during oxidative stress, and conducted detailed analyses of RNA sequencing data from the Gene Expression Omnibus (GEO) database to explore the expression profiles of mRNAs and lncRNAs of LECs under the circumstance of oxidative stress and to try to construct the possible molecular network of ATG7-associated cataractogenic mechanisms, hoping to offer some clues to the studies regarding the prevention and/or treatment of cataracts.

## Materials and methods

### RNA sequencing

Total RNA of human lens epithelial B3 (HLE-B3) cells treated with or without H_2_O_2_ for 12 h was extracted by using TRIzol (Thermo, United States) according to the manufactory’s instructions. After being constructed by using NEBNext^®^ UltraTM RNA Library Prep Kit for Illumina^®^ (NEB, United States) and assessed by the Agilent Bioanalyzer 2,100 system, cDNA libraries were loaded on the Illumina NovaSeq 6,000 platform (Illumina, United States). Each group was sequenced in triplicate.

### Data collection

Raw counts data of genes were extracted from the RNA-seq dataset of GSE161701 in the NCBI’s GEO database ( *https://www.ncbi.nlm.nih.gov/geo/* ). (Huang, Yu, He, He, Yang, Chen and Han, 2022) Specifically, cultured HLE-B3 cells with or without the knockout of *the ATG7* gene were respectively treated with or without 200 μM H_2_O_2_ for 12 h, thus making four groups of samples for subsequent RNA sequencing (i.e., wild type cells for 0 h and 12 h of stimulation (WT-0h and WT-12 h), and *ATG7* knockout cells for 0 h and 12 h of stimulation (KO-0h and KO-12 h), each group with three biological replicates).

### GO and KEGG enrichment analyses

Pearson correlation analysis was first applied to calculate the correlation coefficient between samples and the result was visualized through R package “pheatmap”. Gene expression differences between groups (KO-0h vs. WT-0h, WT-12 h vs. WT-0h, KO-12 h vs. KO-0h, KO-12 h vs. WT-12 h) were detected by using R package “DESeq2” (Love et al., 2014) and genes with adjusted *p*-value < 0.05 and absolute log2 (fold change) > 1 were considered significantly differentially expressed. R packages “ggplot2” (Wickham, 2009), “clusterProfiler” (Wu et al., 2021), “enrichplot”, and “org.Hs.eg.db” were applied for volcano plotting, gene set enrichment analysis (GSEA), and enrichment analysis of gene ontology (GO) (Ashburner et al., 2000; Mi et al., 2019; Consortium, 2021) and Kyoto Encyclopedia of Genes and Genomes (KEGG) (Kanehisa and Goto, 2000). A cutoff of adjusted *p*-value = 0.05 was set for the significance of enrichment analysis.

### Constructions of PPI network and ceRNA network

Protein-protein interaction (PPI) network among the differentially expressed genes was constructed by using the STING database (Szklarczyk et al., 2021) ( *http://string-db.org/* ) and Cytoscape software (Shannon et al., 2003). A densely connected region concerning 10 critical genes from the PPI network was detected *via* the MCODE plugin (Bader and Hogue, 2003) of Cytoscape (Degree cutoff = 2, Node score cutoff = 0.2, K-score = 2, Max. depth = 100). Ten cataract-related miRNAs were collected *via* literature review and the predicted mRNAs and lncRNAs interacted with these miRNAs were obtained by searching the databases of miRDB (Chen and Wang, 2020) ( *http://www.mirdb.org/* ) and Targetscan (McGeary and Lin, 2019) ( *http://www.targetscan.org/* ) and databases of starBase(Li et al., 2014) ( *https://starbase.sysu.edu.cn/* ) and miRnet (Chang et al., 2020) ( *https://www.mirnet.ca/* ), respectively. Then these predicated mRNAs or lncRNAs that were not differentially expressed between the groups (p-adjusted<0.05) were filtered out and a competing endogenouse RNA (ceRNA) network of lncRNA-miRNA-mRNA was plotted *via* Cytoscape software.

## Results

### Gene expression profile of HLE-B3 cells after knockout of *ATG7*

**FIGURE 1:**
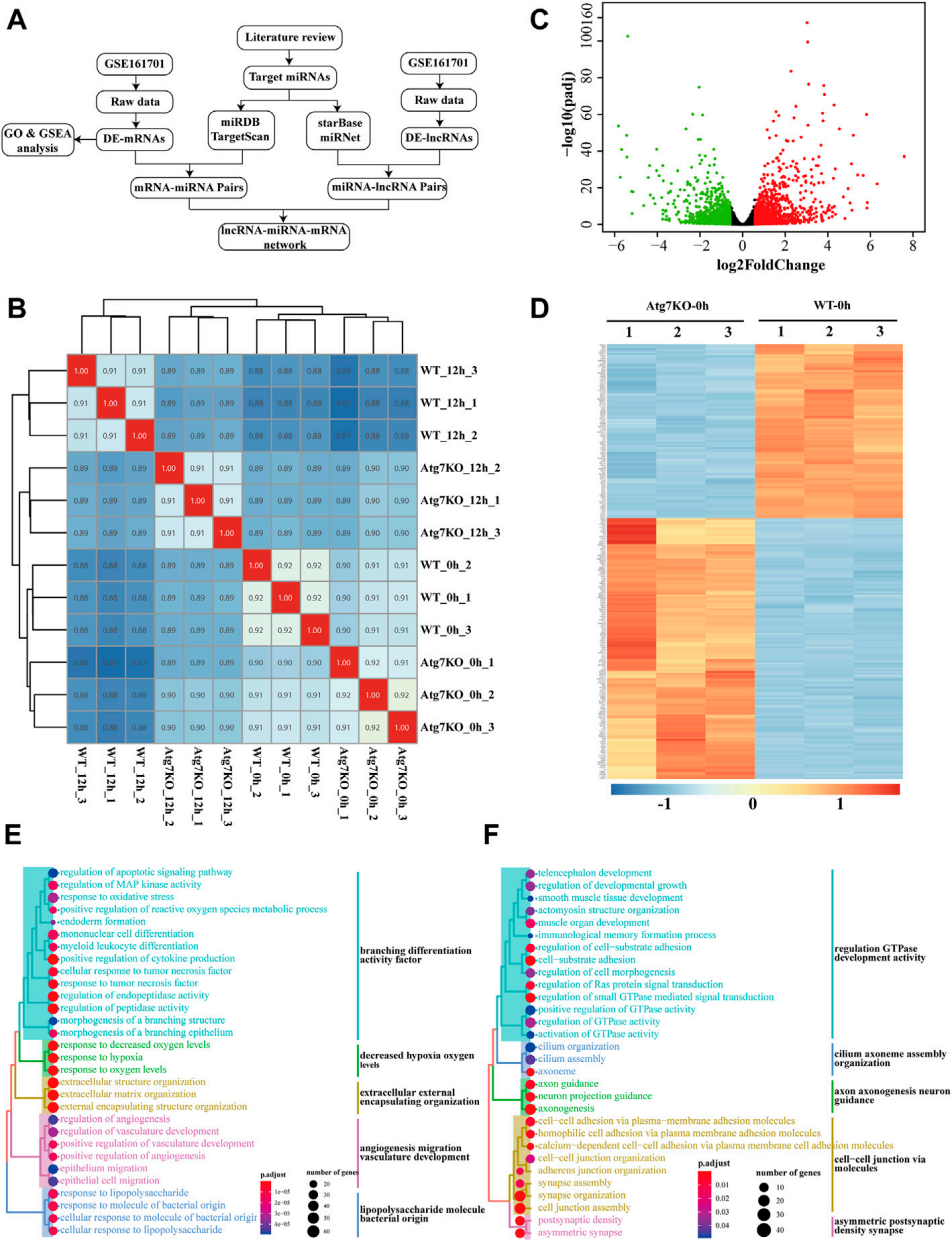
Gene expression profile of HLE-B3 cells after knockout of *ATG7*. **(A)** The flowchart showing the ways the study was carried out **(B)** Heatmap of the sample correlation results. Numbers in the cells denote the corresponding sample correlation coefficients and the blue gradient indicates the degree of coefficients. The darker the color is, the larger the coefficient is. Cells in red indicate the self-correlation. **(C)** Volcano plotting of differentially expressed mRNAs in KO-0h vs. WT-0h groups. Red dots and green dots signify the up-regulated and down-regulated mRNAs. Criteria of fold change >1.5 and adjusted *p*-value<0.05 were applied **(D)** Heatmap of clustered top 250 differentially expressed mRNAs in KO-0h vs. WT-0h groups. The color scale indicates the degrees of expression levels with the blue signifies the most down-regulated and the red the most up-regulated in KO-0h group. **(E and F)** GO enrichment analysis for the differentially up-regulated **(E)** and down-regulated **(F)** genes in KO-0h group. The size of the dots indicates the number of clustered genes and the color of the dots signifies the adjusted *p*-value of enrichment. The enriched GO terms were further clustered in different colors.

First, Pearson correlation analysis was applied to explore the correlations between groups and the result showed that samples treated with H_2_O_2_ were more mutually correlated (Figure 1B), suggesting that H_2_O_2_ treatment exerted more influence on gene expression than *ATG7* knockout. To ascertain the effects of *ATG7* knockout on gene expression, we first performed differential expression analysis of genes between KO-0h and WT-0h groups, and 1,189 up-regulated and 965 down-regulated genes in KO-0h group were found compared with WT-0h group (p-adjusted<0.05 and absolute log2foldchange >1), as illustrated in the volcano plot (Figure 1C). The two group samples can be well clustered by the selected top 250 differentially expressed genes (shown in Figure 1D). To further explore the impacts of *ATG7* knockout, we performed GO analysis for the up-regulated and down-regulated genes, separately. The results showed that the top 30 enriched GO terms for up-regulated genes mainly resided in branching differentiation activity factor, decreased hypoxia oxygen levels, extracellular external encapsulating organization, angiogenesis migration vasculature development and lipopolysaccharide molecule bacterial origin (Figure 1E), while the top 30 GO terms for down-regulated genes were more concerned with regulation GTPase development activity, cilium axoneme assembly organization, axon axonogenesis neuron guidance, cell-cell junction *via* molecules and asymmetric postsynaptic density synapse. (Figure 1F).

### Effects of H_2_O_2_ stimulation on gene expression in HLE-B3 cells

**FIGURE 2:**
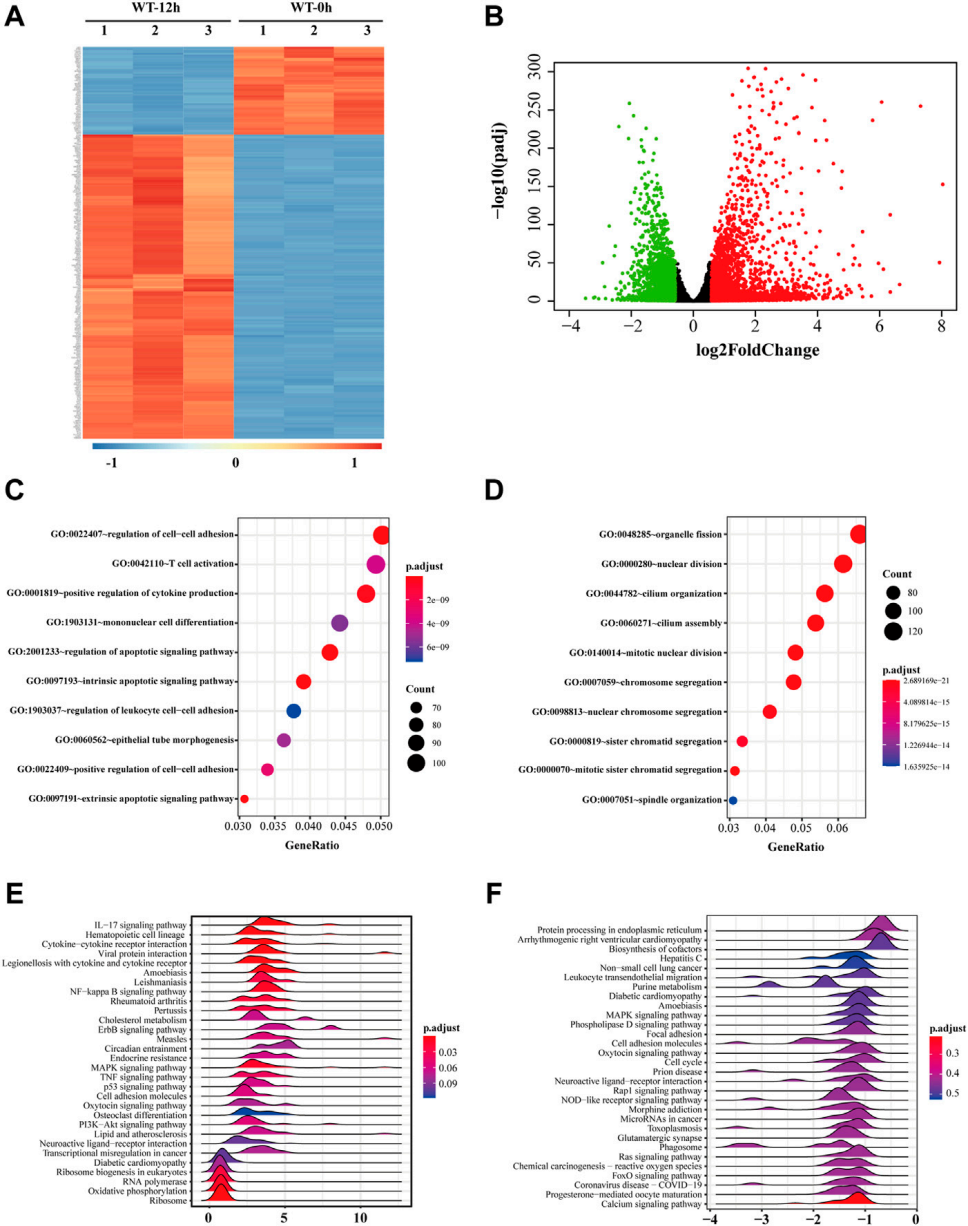
Gene expression profile of HLE-B3 cells after H_2_O_2_ treatment. **(A)** Top 250 differentially expressed mRNAs were clustered based on their expression levels in WT-12 h vs. WT-0h group. The color scale from blue to red is parallel with the expression level from most down-regulated to most up-regulated in WT-12 h group **(B)** Volcano plotting of the distribution of overall differentially expressed mRNAs in WT-12 h vs. WT-0h group. Red dots are for the up-regulated and green dots for the down-regulated. Criteria of fold change >1.5 and adjusted *p*-value<0.05 were applied. **(C and D)** Bubble charts of the top 10 enriched GO terms the up-regulated mRNAs **(C)** and the down-regulated mRNAs **(D)**. The size and color of the dot correspond to the clustered gene number and the related adjusted *p*-value of enrichment. **(E and F)** Ridgeline diagrams show the top 30 KEGG pathways of GSEA for the up-regulated **(E)** and the down-regulated **(F)** mRNAs. *X*-axis indicates the fold change of mRNA expressions. The color of each ridgeline represents the adjusted *p*-value for the enrichment significance of each pathway. GSEA, gene set enrichment analysis.

H_2_O_2_ treatment was applied as a general approach to simulate oxidative stress commonly detected during cataract development. To investigate the biological changes of cultured cells under such oxidative stress, we first analyzed the differential expressed genes after H_2_O_2_ treatment of HLE-B3 cells. A total of 2,384 up-regulated and 2,219 down-regulated genes were finally determined (adjusted *p*-value < 0.05 and absolute fold change >1.5), as demonstrated in the volcano plot (Figure 2B). The top 10 of the up-regulated and down-regulated genes were shown in Table 1. The heatmap further illustrated that these differentially expressed genes can clearly distinguish the WT-12 h group from the WT-0h group (Figure 2A). To further ascertain the possible biological functions of these differentially expressed genes, we performed GO analysis for the up-regulated and down-regulated genes, separately. The results indicated that the top 10 GO terms for up-regulated genes were cell differentiation, regulation of cell-cell adhesion and cytokine production, reproductive associated development, regulation of apoptotic signaling pathway, and intrinsic and extrinsic apoptotic signaling pathway (Figure 2C), while the top10 terms for the down-regulated concerned organelle fission, nuclear division, chromosome segregation, cilium assembly and mitosis related organization (Figure 2D).

**TABLE 1:** The top 10 up- and down-regulated mRNAs in WT 12 h vs. WT 0 h.

mRNA	Log_2_FC	P_adj_	Change
HSPA6	11.59	5.53E-56	UP
AREG	8.03	2.25E-153	UP
CSF3	7.93	4.34E-51	UP
GDF15	7.32	8.91E-256	UP
GREB1	6.64	2.17E-22	UP
LHX3	6.34	1.49E-12	UP
ANGPTL4	6.34	1.28E-113	UP
SH2D2A	6.12	2.16E-42	UP
BTG2	6.07	4.85E-261	UP
PLEKHA6	5.96	4.22E-50	UP
HLA-DRA	−3.47	2.59E-04	DOWN
DACH1	−3.21	5.44E-05	DOWN
CYBB	−3.17	7.82E-06	DOWN
C1QTNF3	−3.05	9.09E-04	DOWN
SIM1	−2.92	7.90E-51	DOWN
PDE7B	−2.86	1.70E-19	DOWN
SORBS2	−2.83	5.36E-04	DOWN
ASPM	−2.81	0.00E+00	DOWN
DIO2	−2.71	7.65E-99	DOWN
MUSK	−2.61	3.86E-02	DOWN

We also performed KEGG analysis *via* GSEA for the up-regulated and down-regulated genes separately to find out the potential pathways these genes involved, and ridgeline plots demonstrated separately the top 30 enriched pathways for the up-regulated and down-regulated (Figures 2E,F). Among them, pathways like hematopoietic cell lineage, NF-kappa B signaling pathway, ErbB signaling pathway, MAPK signaling pathway, TNF signaling pathway, and PI3K-Akt signaling pathway were enriched for the up-regulated while pathways like protein processing in the endoplasmic reticulum, MAPK signaling pathway, oxytocin signaling pathway, rap1 signaling pathway, Ras signaling pathway, and FoxO signaling pathway were enriched for the down-regulated, which suggested these genes under oxidative stress may be involved in cell proliferation and/or apoptosis activities.

### Gene expression differences of two types of HLE-B3 cells under oxidative stress

**FIGURE 3:**
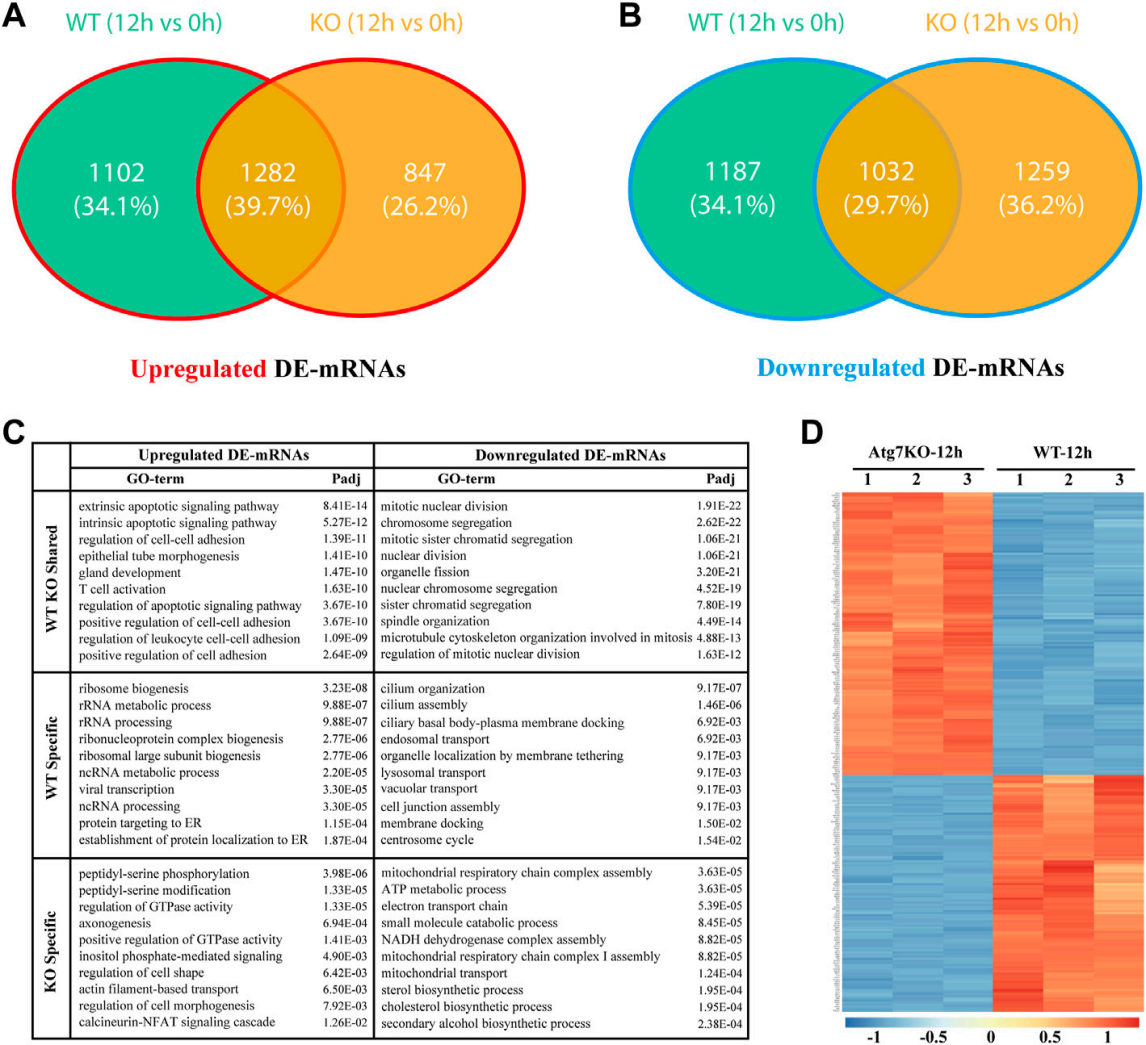
Gene expression differences between wild type and ATG7 knockout HLE-B3 cells under oxidative stress. **(A and B)** The Venn diagrams show the intersected, differentially up-regulated **(A)** and down-regulate **(B)** mRNAs in WT-12 h vs. WT-0h group and KO-12 h vs. KO-0h group, specifically. **(C)** The top 10 GO terms of enrichment analysis for the separate gene parts of the above intersections are summarized in table **(D)** The heatmap of top 250 differentially expressed genes between KO-12 h vs. WT-12 h group. The red and the blue indicate the high and low levels of gene expression, respectively.

To explore whether H_2_O_2_ treatment has the same effects on gene expressions of the two types of cells, we first analyzed the differentially expressed genes in KO-12 h vs. KO-0h groups. Among them, the up-regulated and down-regulated genes were intersected respectively with the up-regulated and the down-regulated ones in WT-12 h vs. WT-0h groups. Results showed that there were 1,282 and 1,032 differentially expressed mRNAs (DE-mRNAs) intersected respectively in the up-regulated and down-regulated groups. These shared genes may be regulated independent of *ATG7* expression under oxidative stress, whereas the genes exclusive to WT-12 h vs. WT-0h group (1,102 in up-regulated DE-mRNAs and 1,187 in down-regulated DE-mRNAs, denoted as green part) may contain the candidates that regulated by *ATG7* (Figures 3A,B). To better understand the functions of parted gene groups, we further performed GO analysis separately for these gene sets. Results indicated that the group-shared gene set of up-regulated DE-mRNAs were enriched mainly in the extrinsic and intrinsic apoptotic signaling pathway, regulation of cell-cell adhesion, and regulation of apoptotic signaling pathway, while the shared gene set of the down-regulated were more enriched in the structural organization involved in mitosis. The enriched GO terms for the up-regulated WT-specific group were mainly related to ribosome biogenesis and ncRNA processing, while the terms for the down-regulated WT-specific group involved cilium organization and assembly, endosomal transport, lysosomal transport, and membrane docking. The KO-specific gene set, however, enriched the GO terms of regulation of GTPase activity, axonogenesis, and regulation of cell morphogenesis for the up-regulated DE-genes and the GO terms concerning ATP metabolic process, small molecule catabolic process, and mitochondrial transport for the down-regulated genes. (Figure 3C).

### Expressions and functions of *ATG7*-related genes in HLE-B3 cells challenged by H_2_O_2_

**FIGURE 4:**
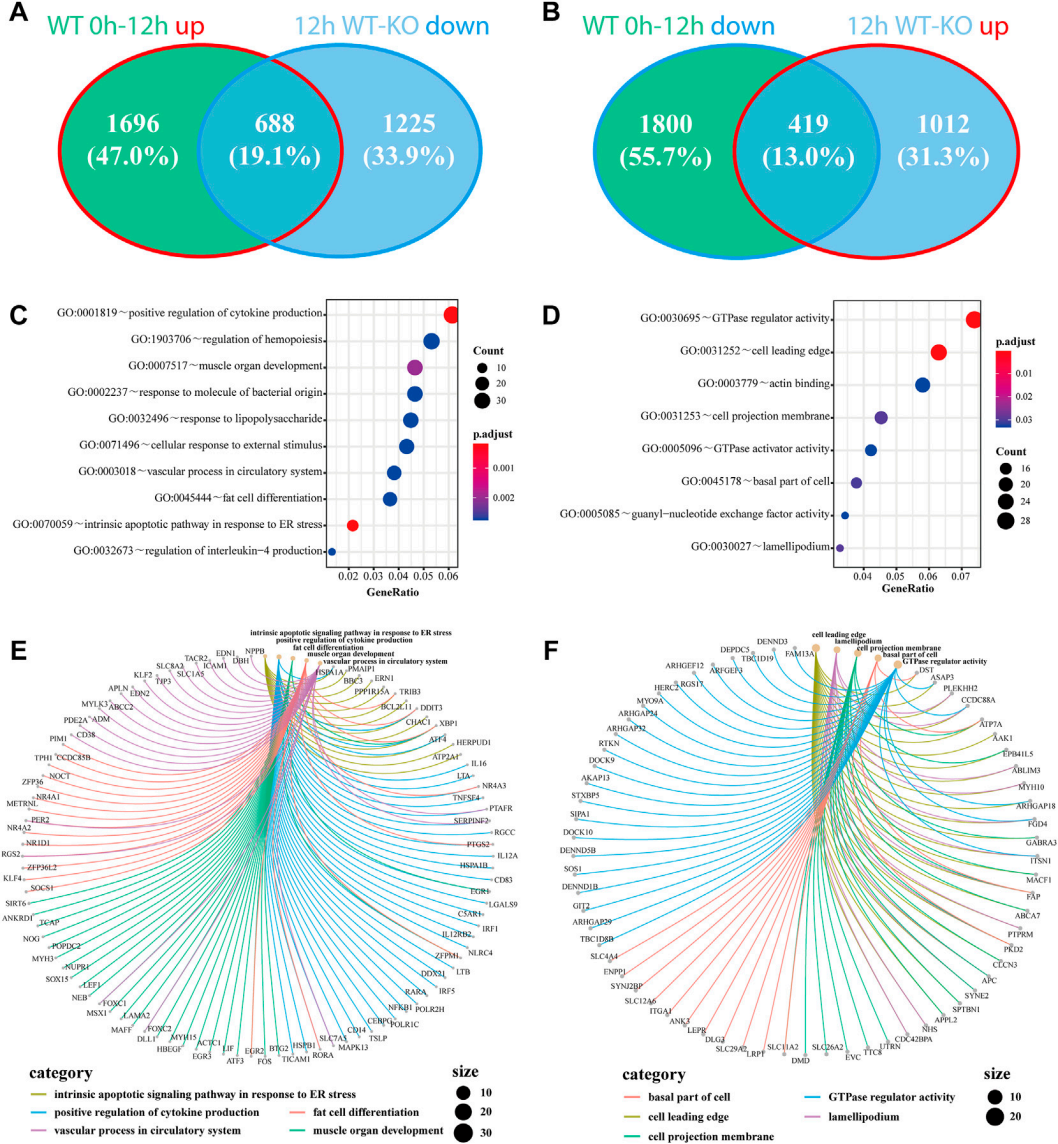
Genes regulated by the knockout of *ATG7* and their associated GO terms under oxidative stress. **(A and B)**The down-regulated genes in 12 h KO-WT group are intersected with the up-regulated genes in WT 12h–0 h group **(A)**, while the up-regulated genes in 12 h KO-WT group are intersected with the down-regulated genes in WT 12h–0 h group **(B)**. **(C and D)** The bubble charts show the top 10 enriched GO terms of the above overlapped parts of differentially expressed genes, separately. C and D correspond to A and B, respectively. Dot size denotes the number of involved genes and color scale represents the adjusted *p*-value of enrichment results **(E and F)** The chord diagrams separately demonstrate the correlations of five representative GO terms with their associated genes. Each color represents a single GO term and dot size signifies the involved gene number.

As a critical gene of autophagy and vacuole transport activity, *ATG7* has been reported to be involved in mitophagy and axonal homeostasis. To further investigate the roles of *ATG7* under the circumstances of H_2_O_2_-induced oxidative stress, we first investigated the differential expressed mRNAs between the KO-12 h group and the WT-12 h group. A total of 1,341 up-regulated and 1912 down-regulated mRNAs were detected based on the criteria of absolute fold change>1.5 and adjusted *p*-value < 0.05, and the top 250 DE-mRNAs can distinctly differentiate the two groups, as demonstrated by the heatmap (Figure 3D). Considering the possible two-way regulations of *ATG7* toward its downstream genes and to narrow down the scope of the downstream genes, the down-regulated genes in the KO-12 h vs. WT-12 h group and the up-regulated genes in the WT-12 h vs. WT-0h group were selected to mutually intersect while on the other hand, the up-regulated genes in the KO-12 h vs. WT-12 h group and the down-regulated genes in the WT-12 h vs. WT-0h group were also selected for another mutual intersection. A Venn diagrams showed that a total of 688 DE-mRNAs possibly positively regulated by *ATG7* and a total number of 419 DE-mRNAs possibly negatively regulated by *ATG7* were finally determined (Figures 4A,B). Next, GO enrichment analysis was performed separately on the above-selected DE-mRNAs. Results showed that enriched GO annotations for the 688 DE-mRNAs included but not limited to intrinsic apoptotic signaling pathway in response to endoplasmic reticulum stress, positive regulation of cytokine production, fat cell differentiation, and regulation of hemopoiesis; while the enriched GO terms for the 419 DE-mRNAs were more about GTPase regulator activity, cell leading edge, cell projection membrane, and organ morphogenesis. The top 10 enriched GO terms were shown in Figure 4C,D. The respective chord diagrams further illustrated the relationships between the five representative GO terms and the annotated input genes (Figures 4E,F).

### Competing endogenous lncRNA-miRNA-mRNA network associated with *ATG7*

**FIGURE 5:**
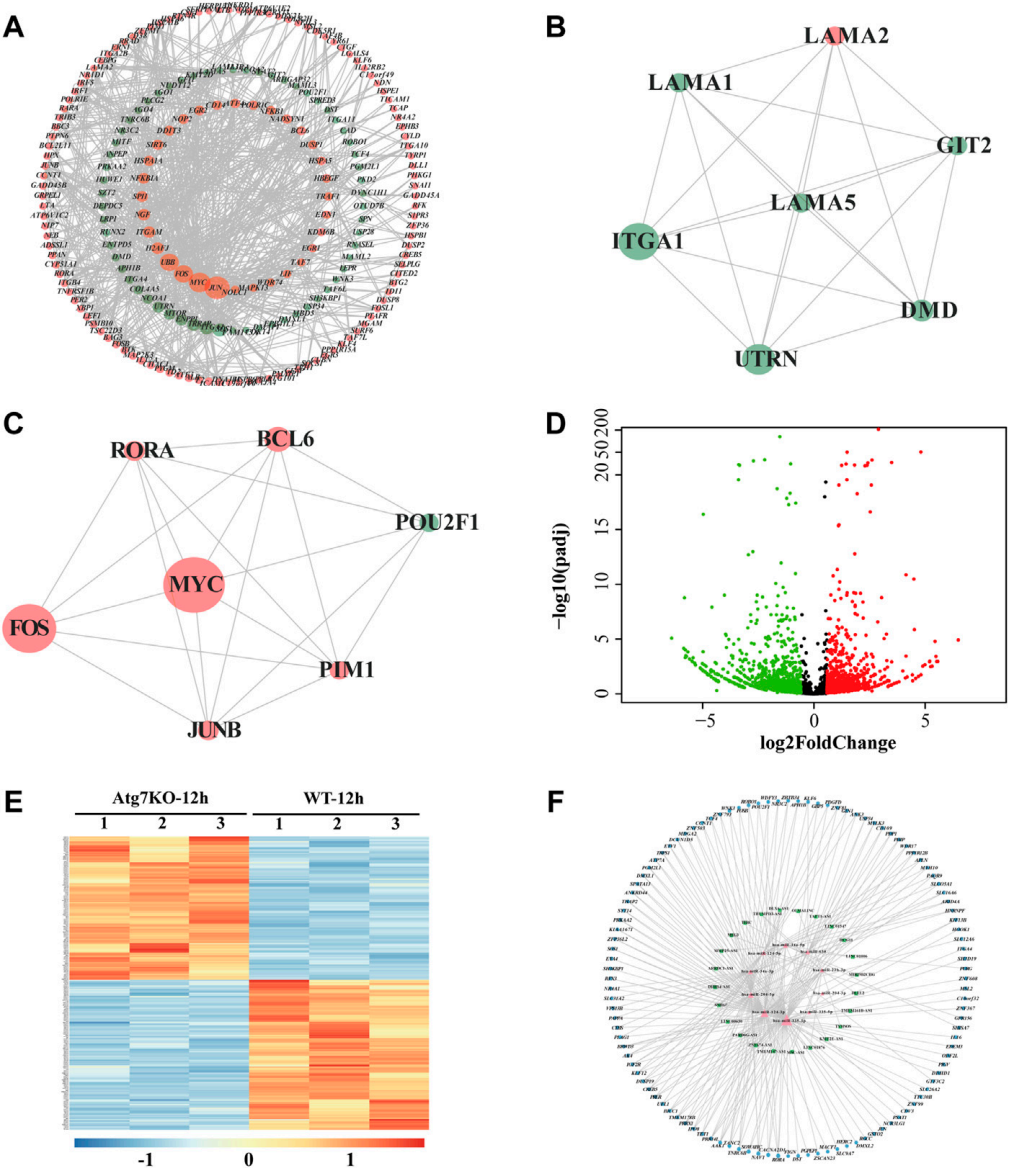
LncRNA-miRNA-mRNA network associated with ATG7. **(A)** PPI network of the intersected genes. Dots in orange represent the overlapped genes in Figure 4A and dots in green for the overlapped genes in Figure 4B. The dot size is in proportion to the number of correlations **(B and C)** Two critical gene modules are detected by the MCODE plugin of Cytoscape. **(D)** The volcano plot shows the differentially expressed lncRNAs in KO-12 h vs. WT-12 h group. Red dots for the up-regulated genes and green for the down-regulated. Criterion include fold change>1.5 and adjusted *p*-value > 0.05 **(E)** The heatmap shows the top 250 differentially expressed lncRNAs in KO-12 h vs. WT-12 h group. Color bar corresponds to the relative expression levels of lncRNAs **(F)** The lncRNA-miRNA-mRNA network constructed *via* Cytoscape. The inner pink triangles represent the selected 10 miRNAs reported involved in cataract development, the green rectangles for the possible competing lncRNAs and the outer blue circles for the targeted genes from the PPI network. PPI, protein-protein interaction.

To further explore the functions of the differentially expressed genes potentially regulated by *ATG7*, we conducted the PPI network analysis *via* the STRING database by using the aforementioned 1,107 (688 + 419) differentially expressed genes as an input list. The result visualized with Cytoscape showed that most of these genes mutually interacted (red dots denote genes from the 688 DE-mRNAs and blue dots represent genes from the 419 DE-mRNAs) (Figure 5A). To find out the downstream key gene module of *ATG7*, we next calculated the interactions within the genes and dug up two key gene modules, each consisting of seven DE-genes *via* the plugin of MCODE (Figures 5B,C). Since non-coding RNAs are emerging as important regulators during cataract development, we also detected the differentially expressed lncRNAs (DE-lncRNAs) in the KO-12 h vs. WT-12 h groups (shown in Figure 5D). There were 263 up-regulated and 336 down-regulated lncRNAs between the groups. The heatmap showed that the top 250 DE-lncRNAs were clustered well in a manner of expression levels between the groups (Figure 5E). The top 10 differentiated mRNAs and lncRNAs were shown in Table 2. Next, through literature review, 10 miRNAs having been reported participating in cataract development were selected and then input into the databases of miRDB and Targetscan and the databases of starBase and miRnet to predict the potential miRNA-mRNA interactions and miRNA-lncRNA interactions, respectively. Those predicted mRNAs and lncRNAs were then intersected with the aforementioned DE-mRNAs and DE-lncRNAs respectively to narrow their scope. Then the more interconnected mRNAs were further selected by the Cytoscape plugin with more strict criteria. Finally, 112 mRNAs and 24 lncRNAs were determined and these RNAs together with the 10 miRNAs were selected to construct the lncRNA-miRNA-mRNA network *via* Cytoscape (Figure 5F).

**TABLE 2:** The top 10 up- and down-regulated mRNAs and lncRNAs in KO 12 h vs. WT 12 h.

mRNA	Log_2_FC	P_adj_	lncRNA	Log_2_FC	P_adj_	Change
AKR1C3	6.59	1.74E-38	**AC006033.2**	6.49	1.20E-05	UP
CPNE8	6.52	2.38E-21	**AC005840.4**	5.57	1.09E-03	UP
LUM	4.93	1.14E-38	**CASC9**	5.52	1.11E-03	UP
KYNU	4.83	5.92E-54	**LINC02328**	5.48	3.83E-04	UP
PARP8	4.53	1.06E-20	**LINC01597**	5.44	1.62E-05	UP
EXOC3L2	4.13	5.14E-17	**AC010931.2**	5.31	2.98E-03	UP
F2RL1	3.96	1.89E-47	**AC006946.2**	5.25	3.25E-03	UP
SOX5	3.83	4.21E-07	**AC103808.3**	5.20	1.05E-03	UP
IL24	3.67	4.53E-06	**LINC01446**	5.19	1.14E-03	UP
TLR4	3.65	4.21E-61	**AC005838.2**	4.99	2.20E-03	UP
BEX1	−7.38	5.05E-145	**AL365181.3**	−6.39	8.12E-06	DOWN
SLC7A5	−7.00	1.84E-67	**AC092813.1**	−5.85	6.97E-05	DOWN
FBLN1	−6.22	1.48E-15	**AL360270.2**	−5.82	1.70E-09	DOWN
HSPA6	−5.25	3.63E-161	**LINC01315**	−5.78	4.78E-04	DOWN
OVGP1	−5.13	6.99E-10	**AC005921.4**	−5.78	9.76E-05	DOWN
GRB7	−4.95	3.71E-05	**AC138356.3**	−5.76	3.16E-04	DOWN
FBLN2	−4.89	1.54E-30	**AC009812.3**	−5.73	3.83E-04	DOWN
H2AFY2	−4.81	6.80E-25	**AC009108.3**	−5.66	1.49E-04	DOWN
NUP210	−4.59	2.76E-86	**TERC**	−5.59	6.50E-04	DOWN
DPYSL4	−4.59	8.00E-109	**LINC01165**	−5.48	1.02E-03	DOWN

## Discussion

Age-related cataract (ARC) is the most prominent type of cataracts and oxidative stress is one of the well-established culprit factors during cataract development, since there is a loss of antioxidants, such as reduced GSH, and increased protein oxidation in the nuclear region of the lens prior to the cataract development. (Giblin, 2000; Truscott, 2005; Vinson, 2006). Based on the location of opacity, ARC can be subdivided into three main types: nuclear, cortical, and posterior subcapsular cataracts. (Liu et al., 2017). Despite their association with aging, the underlying pathological changes and mechanisms seem somewhat different. Nuclear cataract, as the most common type, is more subject to oxidative damage compared with the other two types. (Truscott, 2005). In contrast, the destruction of cell structure and precipitation of soluble proteins are more likely observed in cortical cataract, although it is also associated with some degree of oxidation. As for the less common posterior subcapsular cataract, the failed elongation of swollen fiber cells may be the main cause. When considering the risk factors, there are also differences among the three types of cataracts. Cortical cataract is closely associated with high sunlight exposure and is more commonly seen in high-latitude regions, such as northern Japan and northern China. (Sasaki et al., 2002). Wearing sunglasses is verified as an effective way for its prevention. (Taylor et al., 1988). The posterior subcapsular cataract, however, is more associated with high myopia, diabetes, steroid administration, and ionizing radiation. (Beebe et al., 2010). Such discrepancies among age-related cataracts suggest intricate or even distinct mechanisms underlying cataractogenesis. Therefore, the cell line model stimulated by H_2_O_2_, although commonly used to simulate the factual oxidative stress, can only partly explain the possible mechanisms with caution.

In this study, we detected several pathways and differentially expressed genes that were associated with ATG7. Among them, the intrinsic apoptotic signaling pathway was one of the most enriched pathways for the differentially expressed genes between the groups. As one of the two types of apoptosis signaling, the intrinsic apoptotic signaling pathway is usually activated by internal stimuli such as hypoxia and free radical-induced oxidative stress. It has been found involved in tumor death due to chemotherapy and thus has been investigated as the therapeutic target for drug discovery. (Carneiro and El-Deiry, 2020; Kashyap et al., 2021). With the aging of the lens, lens epithelial cells also underwent certain apoptosis induced by oxidative stress from diabetes, ultraviolet exposure, or just senescence. (Su et al., 2017; Xie et al., 2022). In our study, we detected the intrinsic apoptosis signaling pathway as the potential autophagy-associated mechanism underlying the oxidative stress-induced injury toward LECs. The involved differentially expressed genes in this enriched signaling pathway, such as PMAIP1, BBC3, ENR1, and CHAC1, are thus worthy of further investigation as potential therapeutic targets for cataract development.

It is well recognized that autophagy can contribute to the degradation of damaged or wasted molecules and organelles, including oxidized proteins and lipids, which, if gradually accumulated, could result in cell apoptosis or even necrosis. (Truscott, 2005). The overexpression of *ATG4* can activate autophagy and meanwhile inhibit apoptosis of the HLE-B3 cell line under H_2_O_2_-challenged circumstances. (Yan, Zhao, Qin, Zhao, Ji, and Zhang, 2020). And the rapamycin-induced autophagy can alleviate the ROS production in mice LECs cultured in high glucose. (Liu X. et al, 2020). However, some studies revealed the opposite results. The suppression of autophagy by EphA2 can attenuate the apoptosis of SRA01/04 cells induced by H_2_O_2_. (Han, Wang, Lv, Liu, Dong, Shi and Ji, 2021). Huang J et al. also found that autophagy facilitated the apoptosis of HLE-B3 cells under H_2_O_2_ stimulation. (Huang, Yu, He, He, Yang, Chen and Han, 2022). By mining the RNA sequencing data, our results showed that the differentially down-regulated genes in autophagy-deficient cells are mostly enriched in GO terms related to cell proliferation and differentiation, such as regulation of hemopoiesis, muscle organ development, and fat cell differentiation, which may suggest a pro-apoptotic function of autophagy. The controversial functions of autophagy toward cell apoptosis under oxidative stress may reside in the distinct cell lines and different concentrations and times of H_2_O_2_ stimulation. Further studies are warranted to clarify the relationship between autophagy and apoptosis in a detailed manner.

ATG7, as one of the autophagy-related proteins, is a ubiquitin-activating enzyme E1-like protein and participates, along with ATG3, in the conjugation of ATG8 family proteins to phosphatidylethanolamine (PE) during the phagophore expansion. (Dooley et al., 2014). Although ATG7 is an essential mediator in the canonical autophagy pathway, Atg7-independent autophagy was found in *Atg7*-modified mice, where knockout of *Atg7* did not affect the formation of autophagosome and the subsequent bulk degradation. (Nishida, Arakawa, Fujitani, Yamaguchi, Mizuta, Kanaseki, Komatsu, Otsu, Tsujimoto and Shimizu, 2009). The loss of *Atg7* in *Drosophila* did not prevent the occurrence of autophagy and the accompanied cell size reduction. (Chang et al., 2013). However, such ATG7-independent autophagy has not yet been reported in human cells. Furthermore, ATG components, including ATG7, have found to engage in non-autophagic activities, such as phagocytosis (Sanjuan et al., 2007), osteoclastic bone resorption (DeSelm et al., 2011) and antiviral activity of IFNγ (Hwang et al., 2012). In this study, we found that the DEGs shared by the two types of HLE-B3 cells (KO and WT) under oxidative stress are enriched in apoptotic signaling pathways (Figure 3C), which may suggest an *ATG7*-independent autophagy involved in cell apoptosis.

miRNAs, the highly conserved small ncRNAs across species, have long been found regulating substantial gene expressions by targeting the microRNA response elements (MREs) of mRNAs. (Thomas et al., 2010). Each miRNA can regulate many mRNAs and one mRNA can be regulated by a number of miRNAs. (Friedman et al., 2009). And lncRNA, a group of ncRNAs larger than 200 nucleotides in length, has emerged as an important regulator in a myriad of diseases, including cataract. (Liu, Liu, Shan, Zhang, Lu, Yan, and Luo, 2018; Liu X. et al., 2020; Tu et al., 2020; Shen and Zhou, 2021). On the one hand, they can directly target miRNAs to regulate certain mRNA expressions (Ye and Ma, 2020) while on the other, they can also be targeted by the miRNAs (Chi et al., 2009). Such complicated interactions within the ncRNAs and mRNAs suggest the mechanism of ceRNA. (Salmena et al., 2011). In this article, we constructed a predicated lncRNA-miRNA-mRNA ceRNA network based on the differentially expressed lncRNAs and mRNAs and the reported cataract-related miRNAs. Such a ceRNA network may propose an ATG7-associated potential regulatory mechanism underlying cataractogenesis, which warrants further verification.

## Conclusion

In all, this study reveals the differentially expressed gene profiles of HLE-B3 cells with or without ATG7 knockout subjected to oxidative stress. And by comparing the gene expressions in these two types of cells under oxidative stimulation, we eventually detected differentially expressed 292 mRNAs and 24 lncRNAs that also interacted with the 10 cataract-associated miRNAs. Thus a competing endogenous lncRNA-miRNA-mRNA network was finally constructed based on such interactions, which warrants further investigations.

## Data Availability

The datasets presented in this study can be found in online repositories. The names of the repository/repositories and accession number(s) can be found in the article/Supplementary Material.

## Abbreviations

ARC, age-related cataract; ceRNA, competing endogenouse RNA; DEGs, differentially expressed genes; DE-lncRNAs, differentially expressed lncRNAs; DE-mRNAs, differentially expressed mRNAs; GEO, Gene Expression Omnibus; GGH, glutathione; GO, gene ontology; GSEA, gene set enrichment analysis; HLE-B3, human lens epithelial B3; H2O2, hydrogen peroxide; KEGG, Kyoto Encyclopedia of Genes and Genomes; KO, knockout; LECs, lens epithelial cells; lncRNAs, long non-coding RNAs; MREs, microRNA response elements; ncRNAs, non-coding RNAs; PE, phosphatidylethanolamine; PPI, protein-protein interaction; ROS, reactive oxygen species; rRNAs, ribosomal RNAs; tRNAs, transfer RNAs; WT, wild type.
